# Repercussions of overturning *Roe v. Wade* for women across systems and beyond borders

**DOI:** 10.1186/s12978-022-01490-y

**Published:** 2022-08-24

**Authors:** Karine Coen-Sanchez, Bassey Ebenso, Ieman Mona El-Mowafi, Maria Berghs, Dina Idriss-Wheeler, Sanni Yaya

**Affiliations:** 1 School of Sociological and Anthropological Studies, University of Ottawa, Ottawa, Canada; 2 Leeds Institute Health Sciences, University of Leeds, Leeds, UK; 3 NORImpact, Stavanger, Norway; 4 Unit for the Social Study of Thalassaemia and Sickle Cell, School of Allied Health Sciences, De Montfort University, Leicester, UK; 5 Interdisciplinary School of Health Sciences, University of Ottawa, Ottawa, Canada; 6 School of International Development and Global Studies, Faculty of Social Sciences, University of Ottawa, 120 University Private, Ottawa, ON K1N 6N5 Canada; 7 The George Institute for Global Health, Imperial College London, London, UK

**Keywords:** Roe v Wade, Reproductive health inequities, Abortion, Biopolitics, Social determinants, Reproductive justice

## Abstract

June 24th, 2022, a day that will be etched in today and future generations’ textbooks as a historic day, the United States of America revoked the constitutional right to seek safe abortion care. Overturning Roe v Wade allowed the divided individual states to independently decide the legal parameters regarding abortion care. A decision that disproportionately effects the reproductive lives of women residing on the land of America. Given the systemic impacts of racism, neoliberalism and white supremacy, it is the Black, racialized and poor women who suffer terrible repercussions. In this commentary the authors begin by discussing the historical biopolitical perspective, colonial systems and longstanding impacts on racialized women’s bodies in America. The discussion transitions to the implications of geopolitics at play nationally and cascading impacts globally, focusing on humanitarian and emergency settings. Using a medical humanities perspective, authors highlight the collision between politics and reproductive health policy and its implications on social determinants of health, such as women’s education, employment, housing, racial and gender equity and wellbeing. Long standing advocates, community leaders and healers, leading scientists, birth attendants, doctors, nurses, allied health professionals/providers and humanitarian workers – and many others - are reminded and live the weight of the continuous battle of population control, stemming from the oppressive history of control and exploitation.

## Background

The US Supreme Court’s majority decision to overturn *Roe v. Wade* on June 24th, 2022, has taken away the constitutional right to seek abortion; a decision that will have harmful and inequitable repercussions on the reproductive health of many Americans, particularly Black and Hispanic women in the US. This decision sets the stage for each state to independently decide the legal parameters regarding abortion. According to the Guttmacher Institute, 26 US States have multiple bans ready to enact, 13 of which were trigger laws that automatically took effect when Roe v Wade was revoked, and 11 states had early gestational age bans [1]. Additionally, Justice Samuel Alito’s opinion stated that “they need not even have an exception for incest or rape”, taking away women’s autonomy to make decisions over their bodies [2]. In this editorial, the authors discuss this decision and its impacts on the reproductive health of women, particularly women of colour, through biopolitical, geopolitical and medical humanities perspectives. The idea that “the Constitution makes no reference to abortion” and that this question is to be decided by the people of each state is flawed in both theory and practice. We underscore the injustice of taking away bodily autonomy as well as the disproportionate impacts on already oppressed communities in the US. By women, we mean Black, Indigenous, Women of color, Trans women, and non-binary people who have historically faced, and continue to face, the repercussions of the oppression of white supremacy and the glorification of able white-cis hetero women. We thank those who have come before us to advocate, teach, and heal the wounds in our society.

## Biopolitical perspective on *Roe v. Wade*

Procedures to restrict population growth in “undesirable groups'' in the US has taken place since the nineteenth century. The Eugenics movement focused on eliminating negative traits, a practice aligned with longstanding colonial systems that led to inequities for and harm to Black, Indigenous and racialized American populations, particularly the reproductive health and rights of women [3–5]. Kimberlé Crenshaw's concept of intersectionality illustrates "how racial and gender oppression interact" in the lives of racialized women [6]. Despite these practices now being banned, women of colour still do not have complete autonomy over their bodies and governments have used women as a means to an end rather than as an end in themselves.

As stated by Michel Foucault, biopolitics is a political regulation of the biological aspects of human beings insofar as they were members of given cities, nations or other groupings of people [7]. In the Will to Knowledge (1976), “sexual matters” from the eighteenth century and onward are described as a kind of contact point between a disciplinary anatomy-politics of the human body and regulative biopolitics of the population [8]. Similarly, Giorgio Agamben argues that “the production of a biopolitical body is an original activity of sovereign power”, wherein women are reduced to reproductive life exposed to state intervention [9]. In this sense, the biopolitics of the racialized women’s body is not new. For example, racial capitalism is an example of white supremacy structures, manifested in statements such as “Overturning Roe v. Wade was a victory for “white life” by U.S. Rep. Mary Miller of Illinois, a political declaration that evoked segregation and control over racialized women’s bodies [10, 11]. There is a long and complicated relationship between people of colour and reproductive health in the US; a dismal history of forced birth or forced sterilization of Black and racialized women [4, 5]. These negative experiences continue in the form of systemic racism within the healthcare system. By design, existing healthcare structures create more opportunities for dominant racial groups and reinforce white privilege. Scars from racist policies or medical experimentation on Black people and racialized communities also contribute to a legacy of mistrust. Examples of these are rampant. A case in point, is the Tuskegee Syphilis Study that began in the 1940s in the US where hundreds of Black men with late-stage syphilis were rounded up without prior consent and not offered treatment [12]. There is also the testing of birth control pills by the US on women in Puerto Rico in the 1960s [13, 14]. The Supreme Court’s recent decision only reinforces Foucault’s notion of biopower, allowing government control of reproduction by regulating who gets pregnant and determining who is “fit to reproduce” [15].

Once conceived as a cultural problem for the dominant group—white women—these existing inequalities are reinterpreted as a social problem. The 2022 decision to overturn *Roe v. Wade* has become a global topic of interest for all media outlets, drawing attention to rooted systematic issues that now seemingly apply to ALL women, so the public is taking stock! The history of Black and racialized women receiving forced and non-consensual sterilizations are not a bioethical dilemma; they are the result of longstanding colonial practices that inflict disproportionate, irreparable harm on racialized women. There is a historical dichotomy in the women’s rights movement, as Sojourner Truth, an ex-slave delivered a speech highlighting the lack of inclusivity of Black women in the women’s movement. She questioned the objective to accommodate only the “needs” of white women and asked “Ain't I a woman? [16]. She argued that her claim to equal rights was no less legitimate than those of the white women who were fighting for equal rights to men [17]. This caused a division in the movement, with some Anglo-Saxon women believing that their struggles were superior to those of Black women—perpetuating discrimination and racism experienced by Black women—a failure by white women’s feminist movement to recognize and integrate their anti-slavery consciousness. Historically, racialized women’s bodies were framed by social scientists as something that was welcomed by the victims and their bodies were approached by white men as property [18].

## Political perspective

In overturning Roe-vs Wade, we see the use of political power and the voting process within the Supreme Court to give certain sections of society the power and preference over others, thereby influencing the ability of those sections to make choices about their own reproductive health. A key barrier for access to abortion care in the US is mobility. This is reinforced by the interaction of systemic racism and moral conservatism. Mobility and stasis for Black and women of colour to access safe and legal abortion care nationally will have inequitable and negative financial, social and health effects [19]. Unfathomable hurdles exist for oppressed communities—namely Black and brown women, undocumented immigrant women, refugees and asylum seekers—who need to travel transnationally to access an abortion. As Rafia Zakaria states, “In 2022, opposing abortion is not only sexist, it is also racist”, reaffirming that, educated white women will have access to care, while women of colour carry the brunt of this decision [2]. Expectedly, internal political events in the US often have a cascading effect geopolitically.

The entrenchment of the anti-choice standpoint in the Republican party has undermined access to health care in the US—the same consciousness that has also shaped US foreign policy with impacts on abortion access in what North America refers to as the 'Global South'. The US government has used its power as a leading donor to family planning programmes to pursue policies that conflict with global agreements on reproductive rights including the “global gag rule” that prevents foreign nongovernmental organizations (NGOs) from using their own, non-U.S. funds, to provide abortion services, information, counselling, referrals, or advocacy [20]. Since it was first created in 1984, the policy has historically been enacted by Republican presidents and rescinded by Democratic ones [21]. These decisions have additionally impacted on access to HIV care and support, and increased unwanted/unintended pregnancies and unsafe abortions, all of which led to highly politicized maternal and neonatal mortality and morbidity outcomes continuously monitored by the World Health Organization and the United Nations on the global scale.

Regrettably, NGOs and developing country representatives are not even on the playing field where they can state or discuss how the Global gag rule violates their Global agreement and negatively impact their work in sexual and reproductive health and rights (SRHR) [22]. There is an increased likelihood that NGOs may be deterred from including family planning (FP) in their services given the likely international repercussions of the *Roe v. Wade* decision. Existing fears surrounding funding will be reinforced, resulting in further fragmentation of SRHR and HIV services, staff and trained healthcare provider shortages, increased FP stockouts, and decreased safe abortion commodities. Unfortunately, the funding structure and strength of US’ geopolitical influence on the global stage has implications for strengthening opposition to abortion in governments and civil societies globally.

## Medical humanities perspective

The impact of political power over abortion means greater biopolitical governmentality of women’s bodies and their daily lives in the way that the American state seeks to discipline women and teenage girls [7]. The collision between politics and reproductive health policy has led to a redefinition of women’s ‘personhood’ not only in terms of the ‘foetus’, over questions related to when exactly life begins, but also in terms of people questioning the limits imposed on their self-autonomy and agency over their own relationships and lives. Similarly, the collision between the Supreme Court’s legislative decisions and reproductive health policy has polarised debates in the popular press that may not align with the nuances and complexities about how and why women and their families make decisions about seeking a medical termination or having a surgical abortion. The realities are that abortion is gendered. Abortion impacts on the embodiment of women and girls, care for their physical and psychological wellbeing and involving mainly female healthcare providers and organizations. These realities are further impacted by the Supreme Court legislation and its implications in the 13 Republican-run US states with abortion trigger bans and the ways in which they interpret the law, some in highly restrictive terms that make abortion illegal even in cases of rape and incest [1, 2]. As stated earlier, biopolitics will impact the “North–South” divides, both nationally and internationally, to restrict access to surgical abortion and worsen already existing inequalities of health access. It is yet unclear how this will impact medical abortion (i.e., alternative services such as provision of contraception, the after-morning pill or self-management of abortions at home) [23].

Researchers including Lewandowska (2022) and Guillaume & Rossier (2018) note that in countries where abortions are illegal (Egypt and Jamaica) or permitted only to save a woman’s life (Nigeria and Mexico) and on the basis of health or therapeutic grounds (the Bahamas, Poland and Qatar), women resort to self-management of abortions at home [24, 25]. This raises further questions about how far the State will go in terms of surveillance of women’s bodies to access contraception, essential medicines or services and if or how surveillance technologies will be implemented to impact the services provided by governmental and non-governmental organizations nationally and internationally [25]. People have reported being warned about using social media and/or accessing apps for reproductive services akin to criminalization of abortion and incarceration of Black, Indigenous and racialized communities already affected by inequalities and high rates of deaths [25–27]. Women who cannot gain access to abortions or self-manage abortions at home, and are impacted by socio-economic and socio-cultural inequalities, will potentially give birth to children they do not want and/or cannot care for [28] just as periods of austerity and socio-economic difficulties tend to increase abortions.

The Supreme Court’s decision to overturn *Roe v Wade* will worsen people’s grappling with health-related needs whilst leaving them powerless to solve their reproductive health problems, especially where there is little attention to reproductive justice. People will not have access to abortions if they have ‘miscarriages, ectopic pregnancies, obstetric complications’ [25], if they need ‘lifesaving’ abortions or aftercare [28] and will not have choice over pregnancies (for example if they have cancer or during IVF) [29, 30] nor be able to prepare if their child has significant medical issues/disabilities or if they have a non-viable pregnancy. Taking away that choice to have children or not have children, when to have the children and the ability to nurture the children in a safe and healthy environment, is a clear injustice. Such lack of reproductive justice and choices will once again disproportionately impact Black, Indigenous and racialized women who already have worse maternity and neonatal health outcomes [31], struggle to access essential medical services and are impacted by structural racism across many systems (i.e., education, health, labour). In short, criminalizing abortions nurtures an environment of fear, while stigmatization makes them more deadly as women will pay the price with their lives.

Put differently, the social determinants of health lens [32] facilitates an understanding of how governmentality and lack of reproductive justice in the personal realm, can impact women’s education, employment, and housing and raises ethical quagmires about ensuring gender equity to combat those inequalities. For instance, will the state guarantee affordable nursery care or fund more disability services for those already facing inequalities? The impacts of such governmentality are likely to delay the achievement of many UN sustainable development goals (SDGs) which in the case of women include delaying: Goal 3 of ensuring healthy lives and improving well-being, Goal 4 of ensuring inclusive education and lifelong learning, Goal 5 of achieving gender equality, Goal 8 of promoting full and productive employment for all, and Goal 10 of reducing inequalities with and between countries.

## Conclusion

Taking away the constitutional right to abortion in the US has allowed several states to ban abortion, in turn causing women to not only travel to other states or countries to access abortion but has denied them of choice in their reproductive lives. While the Supreme Court’s decision mainly benefits individuals, organizations and companies who promote, manufacture, distribute and/or sell contraceptive drugs and technologies via globalized supply chains it is a gross reproductive injustice that is not only gendered but is also racialized. Understanding the biopolitical implications of population control, stemming from an oppressive history of control over women, colonization, and slavery, as well as the current US political stage, filled with polarization and racial turmoil, have geopolitical implications globally. The authors acknowledge the hard work of the individuals and groups who have diligently and continuously fought for reproductive health, reproductive rights and reproductive justice for Black, Indigenous, and racialized communities who have been and continue to be marginalized and oppressed. While the magnitude of this Supreme Court ruling will likely accelerate a process that has been evident in recent years, the work is far from over. As the president of the Guttmacher Institute, Dr. Herminia Palacio states, “all of us seeking to defend policies that support bodily autonomy must be ready to meet them [anti-abortion movement] … must protect abortion rights and access in as many states as possible to achieve federal legislation to ensure that anyone, anywhere who needs an abortion can get one freely and with dignity” [1].

## Data Availability

Not applicable.
